# Loneliness and older adults: psychological resilience and technology use during the COVID-19 pandemic—a cross sectional study

**DOI:** 10.3389/fragi.2023.1184386

**Published:** 2023-06-26

**Authors:** Eric Balki, Niall Hayes, Carol Holland

**Affiliations:** ^1^ Centre for Ageing Research, Division of Health Research, Lancaster University, Lancaster, United Kingdom; ^2^ The Directorate, Nottingham Trent University, Nottingham, United Kingdom

**Keywords:** loneliness, psychological resilience, older adults, cross-sectional study, technology

## Abstract

**Introduction:** This study investigated how psychological resilience influenced greater technology use among older adults, and whether they moderated the impact of social isolation on loneliness during the COVID-19 pandemic. We also explored whether technology mediates the impact of psychological resilience on loneliness. To explain the relationship between variables, the research drew upon the socio-emotional selective theory, which posits the notion that older adults are more focused on current and emotionally important relationships and goals concerning emotional regulation goals such as psychological well-being.

**Methods:** Using a cross-sectional observational design, data were collected from 92 residents aged 65 to 89 in England from March 2020 to June 2021. Participants completed the Connor–Davidson Resilience Scale, Technology Experience Questionnaire, UCLA Loneliness Scale, and Lubben Social Network Index. Pearson correlation, mediation and moderation analyses were conducted to investigate the hypotheses.

**Results:** Most participants experienced moderate to severe levels of loneliness, displaying higher levels than pre-pandemic. Psychological resilience predicted greater technology use, and lower levels of loneliness. Technology was found to mediate the relationship between psychological resilience and loneliness. Neither technology use, nor psychological resilience was found to moderate the impact of social isolation on loneliness.

**Discussion:** Findings suggested that strategies directed towards screening older adults for psychological resilience levels and low technology experience may help identify those most at risk for adapting poorly when exposed to stressors in situations like the Covid-19 pandemic. Early interventions can be initiated to increase psychological resilience and technology use, including empirical interventions, that may help decrease loneliness, especially in times of elevated risks for loneliness.

## 1 Introduction

Loneliness is subjective distress resulting from a discrepancy between desired and perceived social relationships (e.g., Perlman and Peplau, 1981) and is associated with depression, anxiety, functional disability and physical symptoms such as pain (Hoogendijk et al., 2020). Globally, older adults were already experiencing high levels of loneliness and social isolation before the pandemic (Berg-Weger and Morley, 2020), with loneliness predicting a range of common health risks, including increased systolic blood pressure (Hawkley and Cacioppo, 2010), infection (Pressman et al., 2005), impaired cognitive function (Wilson et al., 2007), depression (Cacioppo et al., 2010), diminished immunity (Kiecolt-Glaser et al., 1984) and mortality (Brummett et al., 2001). Loneliness is also associated with an increased risk of cognitive decline (Hawkley and Cacioppo, 2010; Ellwardt et al., 2013), dementia (Sutin et al., 2020) and the progression of Alzheimer’s disease (Wilson et al., 2007). It is also known to increase the chances of premature death by 14% (Caballero et al., 2018). Holt-Lunstad et al.’s (2015) observation that social isolation and loneliness is a health risk factor comparable to smoking has been a significantly important message for policy makers and service providers long before the start of the pandemic. Although social isolation and loneliness can exist separately, it is not uncommon for them to coexist, and for social isolation to predict loneliness (Stepanikova et al., 2010).

During the pandemic, the increased risk of older adults contracting COVID-19 and having it progress to a life-threatening state (Ritchie et al., 2020) increased their vulnerability to the disease. Although reducing COVID-19 risk, government mandated social distancing measures potentially worsened the burgeoning problem of social isolation in older adults (Carmen, 2020; Groarke et al., 2020; Van Tilburg et al., 2021; Balki et al., 2022b) and with it, accompanying potential negative outcomes including loneliness. Groarke et al. (2020), whilst studying individuals aged between 18 and 87 between March 23rd and 24 April 2020, showed that disease-containment policies that increase social isolation placed individuals at higher risk of loneliness and continue to do so. In another study by Van Tilburg et al. (2021) on 1,679 Dutch community-dwelling participants aged 65–102 years found that pandemic had increased loneliness. Personal losses, worries about the pandemic, and a decline in trust in societal institutions were associated with increased mental health problems and loneliness (Van Tilburg et al., 2021). Substantial evidence pointed towards an increase in loneliness and its impacts amongst older adults during the pandemic (Emerson, 2020; Kotwal et al., 2021; Krendl & Perry, 2021). This view was mirrored in other studies which found that as physical distancing rules have tightened, rates of loneliness have risen, which may have exacerbated pre-existing mental health conditions (Groarke et al., 2020; Hwang et al., 2020). At its baseline loneliness is associated with worse physical and mental health (Beutal et al., 2017) and increases mortality risk (Holt-Lunstad et al., 2015; Rico-Uribe et al., 2018). Despite the well-established correlations between social isolation, loneliness, disease and mortality and their exacerbation being confirmed during the pandemic, there is a dearth of studies that look at the impact of personal resources the older adults may have used to mitigate the impact of social distancing.

One such personal resource is psychological resilience. Psychological resilience is defined as the process of adapting well in the face of adversity, trauma, tragedy, threats, or significant sources of stress (Sisto et al., 2019). As much as resilience involves “bouncing back” from these difficult experiences, it can also involve profound personal growth (Netuveli et al., 2008). Studies have suggested that although the existence of psychological resilience is universal, it can be thought as a continuum with some people having more resilience than others and it also increasing or decreasing in tandem with situational circumstances (Fletcher and Sarkar, 2013). Psychological resilience can also be considered as either a trait or a process/outcome. As a process, researchers have referred to it as a dynamic process encompassing positive adaptation when facing significant adversity (Luthar et al., 2000). As a trait, psychological resilience represents a constellation of characteristics that enable individuals to adapt to the circumstances they encounter (Connor and Davidson, 2003). During the pandemic, resilience may have had protective effects on the physical and mental status of individuals experiencing or facing adversity and could have impacted loneliness positively (Wortzel et al., 2021). Studies examining resilience in older adults during the pandemic have generally found it to be higher in older adults with Vannini et al. (2021) reporting mean total score for resilience on the CD-RISC-10 questionnaire being 29.5 (based on 141 participants with mean age of 74.4). Scores above 25 are considered to be associated with high average resilience.

The positive impact of psychological resilience on loneliness has been documented in earlier studies (Jakobsen et al., 2020). For example, Gerino et al. (2017) linked loneliness with resilience, mental health, and quality of life in older adults, finding that a high degree of resilience contributed to heighten perceived life quality at the physical and psychological levels and reduced anxiety, depressive symptoms, and loneliness. Although some studies during the pandemic have examined the impact of resilience on loneliness for younger adults (Labrague and Santos, 2020; Marchini et al., 2021), the nexus remains largely unexplored in older adults.

However, other studies during the pandemic found a connection between stress and anxiety and increasing feelings of isolation and loneliness (Balki et al., 2023). Gonçalves et al. (2020) found that when older adults feel worried, particularly about COVID-19, the detrimental effects of social isolation can be amplified on loneliness. Inversely, a sense of being able to successfully adapt to challenging experiences (resilience), can emerge as a potential buffering factor on the impact of social isolation on loneliness (Grossman et al., 2021). These processes and characteristics may have created a defence mechanism in the shape of psychological resilience and against increased social isolation thereby moderating its impact amongst older adults during the pandemic (Patel and Clark-Ginsberg, 2020). Thus, it is clear that its important to take into consideration the impact of resilience on loneliness and social isolation especially in times of elevated stressful conditions such as those imposed as a result of the pandemic.

Another personal resource that older adults could have employed to combat social isolation during the pandemic was technology use. Social support via technology is known to mediate the effects of life stress and loneliness (Pawar and Rathod, 2007; Sippel et al., 2015), but also supports the development of psychological resilience during crisis, and is linked with a reduction in depression, loneliness and an increase in self-esteem (Zautra et al., 2010). If the problem during the pandemic-imposed social distancing measures resulted in deprivation or reduction in social contact, the impact may have also been lessened through use of digital communication technologies (DCT). Videoconferencing apps (such as Teams), instant messaging apps (like WhatsApp) and services (such as Zoom) have grown in popularity during the COVID-19 pandemic, for both business and social activities. It was possible that the use of technology might have mitigated the impact of social isolation, with technologies being used in place of previous in-person visits from friends, family and volunteers. Online group meetings were being orchestrated through videoconferencing programs, as well as religious gatherings like Sunday church gatherings, yoga (Belam, 2020), playing an online game (Nguyen et al., 2017) or new music technology (Court-Jackson, 2011), which all may have decreased feelings of loneliness. Technology use is therefore a logical avenue to investigate as a potential mitigating factor for the impacts of social isolation on older adults but may also have had a potential impact in increasing psychological resilience and through the ability to expand the depth and extent of connectivity (Jurgens and Helsloot, 2018).

It remains possible that technological skills acquired prior to the pandemic were used even more as older adults sought out pathways to remain socially connected through DCT. This investigation aims to explore the relationship between psychological resilience and technology use amongst this demographic and their impact on loneliness levels.

### 1.1 Conceptual framework

To conceptually explain how psychological resilience and technology use could have impacted older adults during the pandemic we drew upon the socio-emotional selectivity theory (SST) and resilience theory. SST argues that as older adult perceive time as more limited (a point that may have been further reinforced by the effect of the pandemic), older adults will value meaningful goals and relationships more than other goals (Kircanski et al., 2016); see also Galindo-Martin et al., 2020). This could have activated mood enhancing goals, as well as making older adults more willing to accept temporary negative experiences such as social distancing, for long term benefits leading to higher psychological resilience to adverse effects. Scheibe and Carstensen (2010), reported similar behaviorism in older adults relating to a shift occurring with age towards more positive disposition. Equally, these factors could have also made older adults seek activities that require technology whilst being socially isolated, like maintaining a connection with loved ones, or a technology enabled mood enhancing activity.

The pandemic can be partly thought of being similar to a natural disaster and may have had similar consequences on older adults. Older adults have been shown to have exhibited higher resilience linked to SST during natural disasters and have more positive disposition than other age groups (Eshel et al., 2016; Rafiey et al., 2016). Other studies have shown that older adults were showing high levels of resilience and coping well during the pandemic strengthening this argument (Fuller and Huseth-Zosel, 2020; Vannini et al., 2021).

Our study aims to provide further empirical support for SST supposition that during the pandemic older adults had high resilience, maintained established relationships especially using technology, and may have provided a degree of protection from the impact of social isolation on loneliness. We use the SST to explain how psychological resilience could have further mediated the relationship between technology and loneliness.

### 1.2 Hypotheses

The following hypotheses provide a basis to investigate these factors:

H_1_. Higher psychological resilience will negatively predict loneliness.

H_2_. Higher psychological resilience will be correlated with greater use of technology.

H_3_. Greater use of technology will reduce loneliness after controlling for the impact of social isolation.

H_4_. Technology will mediate the relationship between psychological resilience and loneliness.

H_5_. Higher psychological resilience and technology will moderate the impact of social isolation on loneliness.

## 2 Material and methods

### 2.1 Study design and setting

This quantitative cross-sectional observational research employed the STROBE checklist (Strengthening the Reporting of Observational Studies in Epidemiology) von Elm et al. (2007). The study was conducted in England starting in 16 March 2020, to 21 June 2021, during the height of the government-mandated COVID-19 social distancing period.

### 2.2 Participants and sampling

A large majority of recruited participants (>80%) were located in the Northwest. The inclusion criteria were older adults (>65) (age inclusion criterion specified by American Psychological Association, 2002); proficient in the English language; and living in their own homes. Older adults living in nursing or care homes, with a history of mental health issues, and who did not speak English, were considered ineligible for this study, due to the variation in ability to participate in this research. Recruitment was conducted through advertisements in senior citizen resource centers, housing associations, third sector organizations, social activity clubs, and local senior groups, via personal approach, and word-of-mouth recommendation. Prospective volunteers telephoned and left a voicemail or sent an email to the researcher, after which a callback determined eligibility.

To determine the minimum size of the research sample necessary for the empirical verification of the tested moderation model, G*Power software (Faul et al., 2009) was used with effect size f2 = 0.15, power = 0.80, and 3 predictors options using multiple regression for the sample size analyses. The total sample size was determined to be 87. A total of 110 volunteers signed up; however, 18 did not complete the questionnaires in their entirety and were excluded. The achieved sample was 92 volunteers aged 65 to 92 (M = 74.6 years, SD = 7.23). All participants identified as either male or female, with more women (n = 55/92, 60%) than men. More than 89% of the participants were White, with less than 11% representing ethnic minorities (n = 7, British Asian, n = 3, British Black). In the Northwest of England, less than 1.4% of the over 65 population is British Black, and less than 6.2% is British Asian (Kings Fund, 2006), and therefore our sample seemed to be representative of areas participants were recruited from.

Sampling ensured a diverse statistically significant representation of the older adult population in England. Due to the difficulties encountered in recruitment during the pandemic and the shrinking time span (to capture maximum effects), we focused on periods when life-space mobility was most restricted.

### 2.3 Variables and measures

Participants first completed a background questionnaire that was developed based on SAGE Encyclopedia of Communication Research Methods (Allen, 2017) as part of a larger study and the variables used in this study included age and ethnicity.

Loneliness was measured using the 20-item UCLA Loneliness Scale (Russell, 1996) with scores ranging from 20 to 80. Higher scores reflected higher loneliness (Cronbach’s alpha = .88).

Technology use was measured using the Technology Experience Questionnaire (Czaja et al., 2006). Participants were presented with a list of technologies (representing communication technology, computer technology, everyday technology, health technology, recreational technology and transportation technology) and were asked to indicate their familiarity with each on a 5-point scale. Scores ranged from 0 to 180 with higher scores indicating greater use and familiarity with technology (Cronbach’s alpha = .84).

Resilience was assessed by the 10-item Connor–Davidson Resilience Scale (CD-RISC-10; Connor and Davidson, 2003), where items were rated on a 5-item scale ranging from 1 (*strongly disagree*) to 5 (*strongly agree*). Questions were described in clear language, for example, “I believe I can achieve goals despite obstacles”, with the surveyor explaining questions where they may not have been understood. The possible scores ranged from 0 to 60 with higher scores reflecting greater resilience (Cronbach’s alpha = .84)

The 12-item Lubben Social Network Index (LSNS-12; Lubben et al., 2006) measured social network size and support, reporting on social isolation levels. The possible score range was 0–60 with a higher score indicating more social engagement and greater social connectedness (Cronbach’s alpha = .88).

### 2.4 Ethics

Ethical procedures aligned with the British Psychological Society guidelines. The study received ethical approval from the University Faculty Research Ethics Committee (Ref: FHMREC19121). Participants were provided with an information sheet and allowed to ask any questions. They were informed of their rights to withdraw at any point in the research and advice about anonymity. Their consent was given either via email or read over the telephone. Data were captured over the phone after the identity of the participant was confirmed, recorded in spreadsheets and anonymized thereafter.

### 2.5 Procedure

Telephone surveys collected information on loneliness, social isolation, technology use and psychological resilience in addition to basic demographic information. Google Analytics was used to record and tabulate the data, with further analysis done using IBM SPSS Ver 28. Participants completed the assessments across 14 months, spanning various levels of COVID-19-related lockdown measures.

### 2.6 Statistical methods

All analyses were carried out using 95% probability. There were no missing data identified among the observations obtained. The variables of loneliness, technology use, social isolation and psychological resilience were screened for skewness and kurtosis to assess the deviation of their distributions from normality using a histogram with simulated overlapping normal curves. The homoscedasticity of the residuals was checked using a standardized residual versus a standardized predicted plot. Mahalanobis (*p* < 0.001) and Cook’s distance were used to check for a linear relationship between dependent and each independent variable using a scatterplot matrix of dependent and continuous independent variables to establish if there were any high leverage points, significant outliers, or highly influential points. Before removing any significant outliers, a linear regression was performed to check the variance caused by the data point included and if it needed to be removed from the dataset. The criteria for discarding observations were the inability to meet two of the three gauges of the distance measures used. However, no outliers were found that would significantly impact the findings, and thus, none were removed. Confirmation of independence of observations and the assumption of no autocorrelation in residuals was checked using the Durbin-Watson d-statistic.

Initial descriptive analyses included frequencies, means and standard deviations. Pearson product-moment correlation coefficients were calculated to determine if there was an association between dependent and continuous variables, whether higher psychological resilience predicted lower loneliness (Hypothesis 1) and greater use of technology (Hypothesis 2).

Multiple linear regression models were built to evaluate whether greater technology use predicts reduced loneliness after controlling for social isolation to examine Hypothesis 3. The associated predictor variables were entered into the model and a backward elimination approach was used, removing any variable with α > 0.15 (here α is defined as the critical *p*-value).

To test whether psychological resilience mediates the relationship between technology use and loneliness (Hypothesis 4), we used Hayes’ PROCESS macro (v3.2) (Hayes, 2017) Model 4, which allows testing the mediating relationship with bootstrap confidence intervals (CIs) for an indirect effect. We applied a bootstrapping approach to determine the indirect effect for each of the bootstrapped 5000 sample items from the original dataset using stochastic sampling with replacement. As a nonparametric resampling procedure, bootstrapping is considered the most powerful method for small samples because it is the least vulnerable to Type I errors. If the CIs did not include zero, then the effects were significant (*p* < .05).

Hayes’s (2017) PROCESS macro for SPSS with Model 1 was applied to investigate the moderating effects of psychological resilience and technology use on social isolation for loneliness as per Hypothesis 5. Moderation effects were examined by comparing the stratified models using Z-scores. If the standardized coefficients of the interaction terms were significant (*p* < .05) or marginally significant (*p* < .09), we conducted a simple slope test to examine the interaction effect at different levels to explain the moderating effect further.

## 3 Results

Table 1 shows the results for calculated means and standard deviations, maximum and minimum as basic descriptive statistics.

**TABLE 1:** Descriptive statistics (*N = 92*).

Scale	Minimum	Maximum	*M*	*SD*
UCLA-Loneliness Score	20	80	47.49	17.814
LSNS-12	1	49	26.91	15.304
CD-RISC-10	5	36	21.76	10.543
Technology Experience	48	175	116.87	40.951

Participants demonstrated moderate to high levels of loneliness with 44% of older adults demonstrating loneliness scores of above 50 (Russell, 1996). The Lubbens Social Network Scale (LSNS-12) indicated that the majority of participants reported good levels of social connectedness with 82% scoring above 25. For psychological resilience (CD-RISC-10), most participants (>57%) scored above 25. As far as technology use was concerned, most participants scored above 125 (56%), demonstrating high use and familiarity with technology in general (Czaja et al., 2006). However, we did find that a significant number of participants (32%) scored below 120, which indicated low familiarity and use of technology (Czaja et al., 2006) and a binormal distribution.

Pearson correlation coefficients were conducted to establish the relationship between loneliness, technology, and social isolation. A correlation matrix of the variables was examined to investigate hypotheses 1 and 2. Table 2 presents the results from the correlational analysis.

**TABLE 2:** Correlational analysis between variables (*N = 92*).

		UCLA- loneliness score	LSNS-12	CD-RISC-10	Technology experience
UCLA-Loneliness Score	Pearson correlation	1	−.853^**^	−.885^**^	−.631^**^
LSNS-12	Pearson correlation	−.853^**^	1	.866^**^	.557^**^
CD-RISC-10	Pearson correlation	−.885^**^	.866^**^	1	.610^**^
Technology Experience	Pearson correlation	−.631^**^	.557^**^	.610^**^	1

**p < 0.01 (two-tailed).

Hypothesis 1: The correlational relationship between psychological resilience and loneliness

Table 2 shows that the correlation between psychological resilience and loneliness score was statistically significant (r = −0.885, *p* < 0.001) and negatively correlated. This meant that higher psychological resilience correlated with lower loneliness scores, thus supporting our first hypothesis.

Hypothesis 2: Higher psychological resilience is correlated to greater technology experience

Table 2 shows that the correlation between psychological resilience and technology experience was statistically significant (r = .610, *p* < 0.001) and positively correlated. This meant that higher psychological resilience was correlated with higher levels of technology use during the pandemic, thus supporting our fourth hypothesis.

Hypothesis 3: Greater use of technology predicts lower loneliness, after controlling for social isolation

Estimates regarding the residual of the hierarchical multiple regression model on loneliness were checked and found to follow a normal distribution. Analysis examining whether greater technology use level predicts lower loneliness after controlling for social isolation (Hypothesis 3) was conducted using hierarchical multiple linear regression analysis. Loneliness was set as the dependent variable, technology experience as independent variable and social isolation as the control variable. Table 3 shows the coefficient results of the multiple regression analysis.

Results showed that both technology experience (b = −0.098, t = −3.645, *p* < 0.001) and social isolation (b = −0.847, t = −11.727, *p* < 0.001) were significant negative predictors of loneliness, i.e., higher social connectedness and greater technology use was linked to lower loneliness scores. The results of the ANOVA test for the significance of the regression models showed that the combined effect for both predictors was significant (F (2, 89) = 143.721, *p* < 0.001). Adding social isolation to the multiple regression model changes the value of R^2^ by 0.035 (*p* < 0.001). Therefore, technology experience significantly predicted loneliness score after controlling for social isolation, thus confirming our second hypothesis.

**TABLE 3:** Model output and coefficients of multiple linear regression model on loneliness.

Model	Regression equation		Overall fit				Significance of regression coefficient	
	Dependent variable	Independent variable	R	R^2^	Δ R^2^	F	Β	t
Model 1	Loneliness		0.853	0.728		241.216		
		Intercept					74.224	37.533***
		Social Isolation					−0.993	−15.531***
Model 2	Loneliness		0.874	0.764	0.035	143.72		
		Intercept					81.778	29.403***
		Social Isolation					−0.847	−11.727***
		Technology Experience					−0.098	−3.645***

∗∗∗*p* < 0.001.

Hypothesis 4: The mediating role of technology use between psychological resilience and loneliness.

**FIGURE 1:**
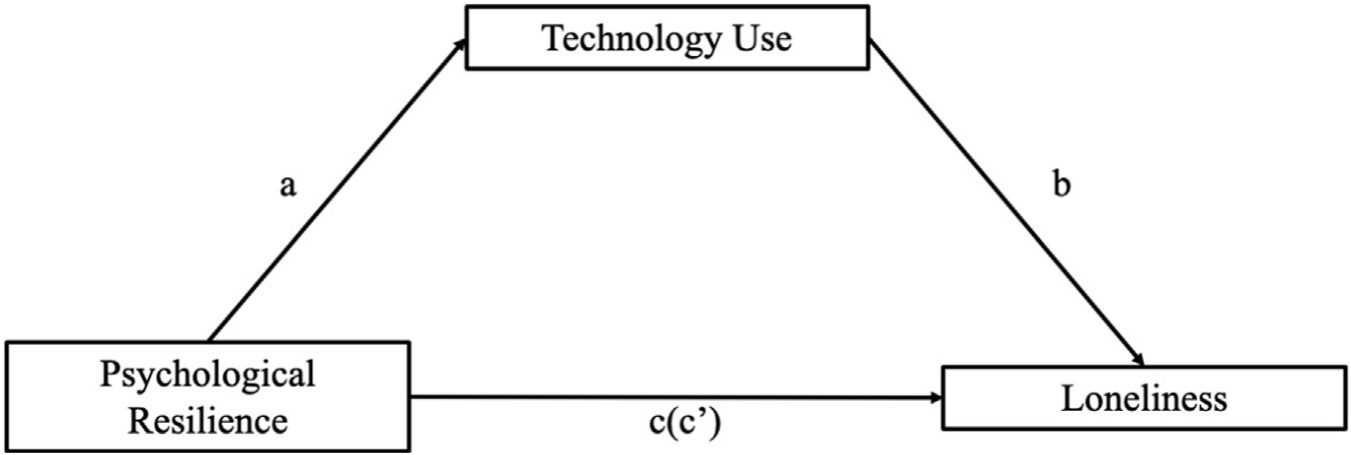
The mediating role of technology use between psychological resilience and loneliness. Here, a represents the effect of psychological resilience on technology use, b represents the effect technology use on loneliness, c represents the total effect of psychological resilience on loneliness. c’ represents the direct effect of psychological resilience on loneliness.

Using Model 4 in SPSS’s PROCESS macro40 compiled by Hayes (2012), we tested the mediating effect of technology use in the relationship between psychological resilience and loneliness, with the results summarized in Table 6 and seen in Figure 1.

Table 4 shows that psychological resilience had a significant predictive effect on loneliness (path c) (B = −0.88, t = −18.0254, *p* < 0.001), and when technology use (the intermediary variable) was put in, the direct predictive effect of psychological resilience on loneliness (path c’) was still significant (B = −0.80, t = −13.1933, *p* < 0.001), indicating incomplete mediation. The positive predictive effects of psychological resilience on technology use (path a) (B = 0.61, t = 7.298, *p* < 0.001) and negative effects of technology use on loneliness (path b) (B = −0.15, t = −2.4139, *p* < 0.05) were also significant. Boot strap mediation was tested where Boot LLCI and Boot ULCI are 95% confidence limits. If the 95% confidence limit includes zero, the indirect effect test is not significant. The direct effect of psychological resilience on loneliness was established (upper and lower limits of bootstrap at the 95% confidence interval [−1.55 −1.14] did not contain 0), while the mediating effect of technology use was not significant (upper and lower limits of bootstrap at the 95% confidence interval [−0.42 0.04] contained 0); the bootstrapped mediation indicated that technology use only partially mediated the relationship between psychological resilience and loneliness as shown in Table 5.

**TABLE 4:** Intermediary model test of technology use.

Regression equation		Overall fit			Significance of regression coefficient	
Dependent variable	Independent variable	*R*	*R* ^*2*^	F	Β	t
Loneliness		0.88	0.78	324.9142		
	Psychological Resilience (c)				−0.8849	−18.0254***
Technology Use		0.61	0.37	53.2609		
	Psychological Resilience (a)				0.6097	7.2980***
Loneliness		0.89	0.80	174.084		
	Technology Use (b)				−0.1457	−2.4139*
	Psychological Resilience (c’)				−0.7961	−13.1933***

∗*p* < 0.05. ∗∗*p* < 0.01. ∗∗∗*p* < 0.001.

Note: All the variables in the model are standardised and brought into the regression equation.

**TABLE 5:** Decomposition table of total effect, direct effect, and indirect effect.

	Effect	Boot SE	Boot LLCI	Boot ULCI	Relative effect value (%)
Total effect	−1.4953	0.0830	−1.6601	−1.3305	
Direct effect	−1.3452	0.1020	−1.5478	−1.1426	89.96
Indirect effect	−0.1501	0.1193	−0.4187	0.0409	10.04

This intermediary effect accounted for 10.04% of the total effect.

Hypothesis 5: Psychological resilience and technology experience will moderate the impact of social isolation on loneliness.

**FIGURE 2:**
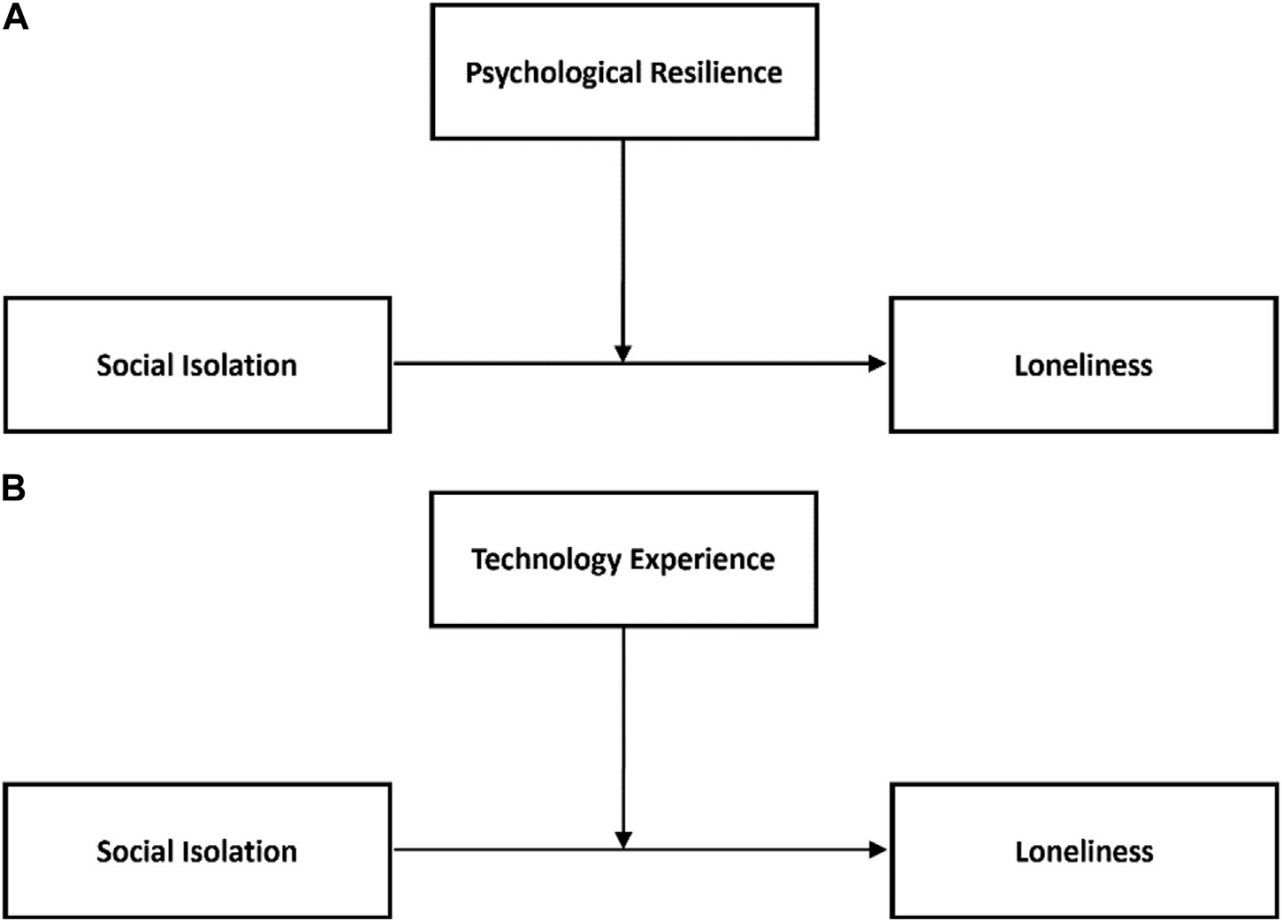
**(A)** The moderating role of psychological resilience on social isolation. **(B)** The moderating role of technology experience on social isolation.

We conducted the test of this hypothesis in two subsections. Model 2 was used in the PROCESS 4.0 macro for SPSS to examine the moderation effect of psychological resilience on social isolation for loneliness first, followed by moderation effect of technology experience on the relationship between social isolation and loneliness as proposed in Hypothesis 5 (Hayes 2018) and as presented in Figures 2A,B.

Here, all continuous variables were converted to Z-scores for use in the model. Z-scores describe the position of raw scores in terms of their distance from the mean, when measured in standard deviation units and standardize the distribution. Table 6 shows that the unconditional interaction (unconditional interaction looks at mean interaction variables) of social isolation and psychological resilience was not significant (β = 0.07, t = 0.8, *p* > 0.05).

**FIGURE 3:**
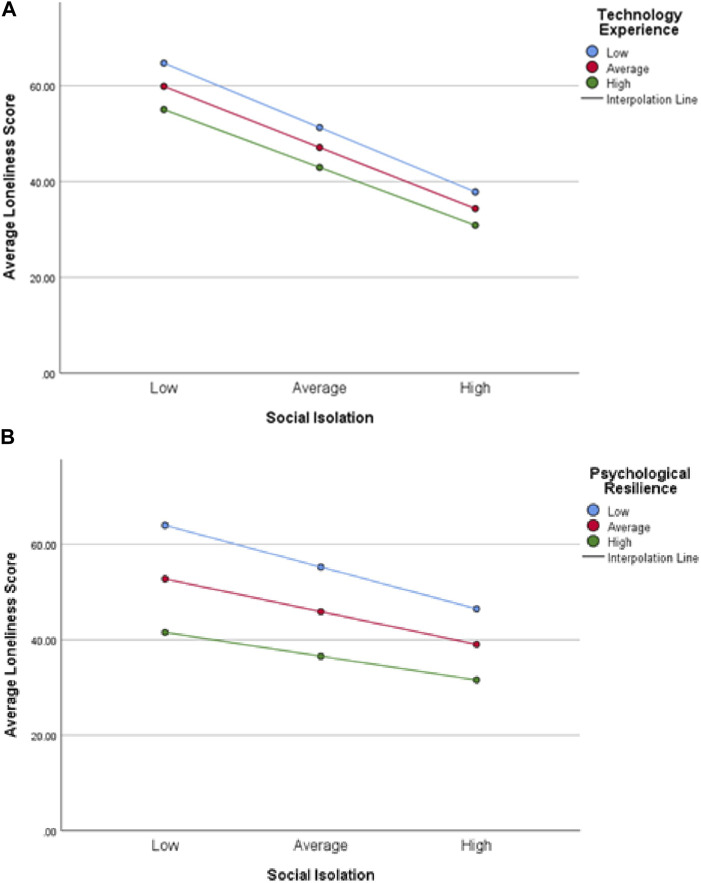
**(A)** Moderating effect of psychological resilience on social isolation. **(B)** Moderating effect of technology experience on social isolation.

We also saw that unconditional interaction of social isolation and technology use was not significant (β = −0.01, t = −0.67, *p* > 0.05) either. To check whether there was any conditional interaction between the variables, we carried out a simple slope test as can be seen in Figures 3A,B.

When psychological resilience was low, social isolation and loneliness were also not significantly correlated (βsimple(M-1SD) = 0.134, *p* > 0.05). Moreover, when the psychological resilience was high, social isolation and loneliness were not significantly correlated (βsimple(M+1SD) = 0.11, *p* > 0.05). This is visible in Figure 3A, with all three slopes parallel to each other. Next, when the technology experience was low, social isolation and loneliness were not found to be significantly correlated (βsimple(M-1SD) = 0.057, *p* > 0.05). Moreover, when the technology experience was high, social isolation and loneliness were again not significantly correlated (βsimple(M+1SD) = 0.046, *p* > 0.05). This can also be noted by the fact that all three slopes are parallel to each other in Figure 3B. Thus, we can conclude that higher psychological resilience and technology use do not significantly moderate the impact of social isolation on loneliness. Therefore, our fifth and final hypothesis was rejected.

**TABLE 6:** Moderation analysis for the effect of social isolation on loneliness with technology use and psychological resilience as moderators.

	Overall fit indicators			Significance of standardized coefficient	
Independent variable	*R*	*R* ^*2*^	*F*	*β*	*T*
	0.91	0.83	81.60		
Social Isolation (ZSN1)				−0.36	−3.73***
Psychological Resilience (ZPR1)				−0.50	−4.67***
ZSN1*ZPR1				0.07	0.8
Technology Experience (ZTE1)				−0.11	−1.83
ZSN1*ZTE1				−0.01	−0.67

*Note*: *** *p*< .001.

Note: All the variables in the model are standardized and brought into the regression equation.

## 4 Discussion

This study set out to examine the role played by psychological resilience and technology use on loneliness levels of older adults during the COVID-19 pandemic. We explored the mediating role played by technology in the relationship between psychological resilience and loneliness. We also explored whether psychological resilience was correlated with higher technology use and whether it played a moderating role in the effect of social isolation on loneliness. We also explored the relationship of technology experience with psychological resilience.

Our study found higher levels of loneliness during the height of the Covid-19 pandemic, especially when compared to pre-pandemic data. Victor and Yang. (2012) loneliness levels showed 30% on average, while Hawkley et al.’s (2020) comparison of loneliness across two continents found the prevalence to be around 25% in older adults compared to the 44% in this study using the UCLA loneliness scale. This confirms the heightened occurrence of loneliness prevalence during the pandemic and concurs with several studies that found a similar condition (Bu et al., 2020; Elran-Barak and Mozeikov, 2020; Emerson, 2020; Krendl and Perry, 2021).

The ability to use technology successfully to adapt to challenging experiences during lockdown emerged as a potential factor to reduce loneliness. Higher technology use was associated with lower loneliness. Having the ability to adapt to challenging experiences (psychological resilience) also predicted lower levels of loneliness. Psychological resilience was correlated to higher technology use, which seems to indicate that resilience may be playing role in increased use of technology during the pandemic. This is a notable finding and could potentially be because of perseverance and better coping attitudes associated with higher psychological resilience. The SST theory posited an explanation that older adults in times of stress could be seeking access to information that equipped their coping mechanisms better, using technological means, communicating with friends and family. This information could have also given them ability to cope with stressful situations but also potentially the ability to learn and persist with using technological tools, a point that has been alluded to in previous studies pointing to a bidirectional relationship (Bustinza et al., 2019). In a recent study by Savitsky et al. (2020), higher levels of resilience and positive coping skills related to decreased levels of pandemic related anxiety among participants during government mandated social distancing. The relationship between technology experience and psychological resilience appears to be complex, bidirectional and needs further in-depth research. It should be noted that the lack of pre-pandemic data on participants precludes any certainty that the justification for the results is valid.

Technology use was found to mediate the relationship only partially between psychological resilience and loneliness (Hypothesis 3). This was a notable observation as individually both psychological resilience and technology use were found to have an unconditional direct impact on loneliness levels. Technology could have an impact on helping older adults in finding new and effective pathways to connect with others and access information that would have mitigated thoughts that enhance loneliness. Systematic reviews across disciplines have conceptualized psychological resilience as encompassing multiple components including: a personal characteristic shaped by social contexts, a dynamic and agentic process of adapting to challenges or stressors, and an outcome that is favorable in spite of adversity or trauma (Hjemdal et al., 2006; Windle, 2011; Smith and Hayslip, 2012). However, technology could have also given access to negative information regarding the pandemic to older adults thereby neutralizing any mediation impact of technology.

Neither psychological resilience nor technology use moderated the impact of social isolation on loneliness. We can conjecture the reasons why this was possible, and this may be down to older adults having access to information via technology that perhaps led to more negative feelings towards being isolated, or perhaps worsened their isolation, increased anxiety and/or stress, or simply had little to no impact. Mustafa and Gold (2013) presented the concept of a sense of technology enabling a “perpetual contact” with a sense of being “chained,” never being able to escape the demands imposed by a persons social network. It is more likely that the latter explanation was more plausible as the plethora of information that increased anxiety related to the Covid-19 pandemic as has been reported in recent studies (Drouin et al., 2020; Balki et al., 2022a; Balki et al., 2023), would have impacted psychological resilience negatively as stress and anxiety have been found to be negatively correlated to psychological resilience (Bitsika et al., 2013).

When linked to the socio-emotional selectivity theory, the pandemic may have influenced older adults to achieve their emotional goals (Carstensen, 1992), bringing them together in positive as well as negative ways. In coping with loneliness, older adults improving relationships through technology means investing in existing contacts and being pre-disposed to positive or negative outlooks.

Our results confirmed the correlation between technology use and lower levels of loneliness. Studies have previously highlighted the potential positive effects of technology use on individual wellbeing by decreasing the likelihood of social isolation due to the increase in connectivity, a sense of belonging, and a decrease in loneliness (Burke et al., 2010; Stepanikova et al., 2010). Technology can increase the potential of expression that is often limited in daily interactions (Rosenberg, 2019) and especially important in times of crisis (Chan, 2013). Technology can also increase the likelihood of positive social support from social groups, family, friendships, and community that are especially important when someone is disconnected from the external environment, as experienced by older adults in our study in a COVID-19 lockdown situation (Dolev-Cohen and Barak, 2013). The concept of social support is especially pertinent because it mediates the effects of life stress on health and wellbeing (Sippel et al., 2015). Positive social support can protect against stress and facilitate the development of psychological resilience among individuals facing significant adversity (Zautra et al., 2010).

Older adults using technology for “social exchange” may communicate more frequently to exchange information and opinions, such as on the downsides of the pandemic. However, these prompt, spontaneous, and intentional or accidental increases of technology-mediated dialogue could potentially worsen social isolation. The motivation for this behavior occurs when individuals seek to increase and maintain high personal involvement in matters of significance as explained by the social contagion theory (Heath and Porter, 2017). The dual positive and negative effect of technology makes it a complex moderating or mediating variable, and its impact of it in conjunction with psychological resilience needs further investigation. For example, certain information-based technology application (such as SNS) may cause a negative impact, as opposed to others that are purely enable communication (such as videoconferencing).

Psychological resilience and adequate coping skills have been identified as vital personal resources to effectively manage and rebound from stressful situations such as disease outbreaks and disasters (Duncan, 2020). Earlier studies involving the general population have linked psychological resilience to reduced anxiety, stress and depression (Labrague and Santos, 2020) and improved mental and psychological health (Cooper et al., 2020). This study confirmed resilience as a protective factor against loneliness mitigating other negative effects of social distancing and lockdown measures during the pandemic (Groarke et al., 2020).

Technological advancements offer remarkable opportunities for older adults to maintain connections despite the need to stay physically separated. Further, although psychological resilience has a complex but important role to play in alleviating loneliness and greater technology use, there is a need to help people build psychological resilience. Beyond achieving mastery, the key to alleviating loneliness is to encourage a more positive outlook on technology use. Psychological resilience can be perceived as a dynamic, adaptive process that has important implications for cultivating and maintaining health and wellbeing in later life. Resilience can be taught and learned (Manning, 2013), and interventions that help individuals build resilience as a distal resource could have important, long-term effects.

## 5 Limitations

The study had several limitations, although these did not distract from the potential importance of the findings in relation to understanding how loneliness could be prevented in a time of adversity. First, the data produced through this research design cannot determine causality. Directionality may be important when examining loneliness in the context of resilience as earlier studies suggest that being in a restrained environment contributes to a resilient reaction (Sygit-Kowalkowska et al., 2017). Therefore, a longitudinal study would allow for generalizability, while a mixed-method approach would include the voices of the affected population. Second, the sampling bias was skewed toward populations with access to and literate in digital resources, or those who were more socially connected via virtual platforms. This is because most study participants were recruited through technology, including mobile phones and email. Their ability to manage the technology suggests that such participants may have experienced less loneliness.

Future studies might clarify this issue, as it may be possible to collect more detailed measures in order to receive more accurate data. In addition, the absence of pre-pandemic data precludes comparison with the pre-pandemic measures.

We also found that psychological resilience played a complex interactive role with technology and loneliness, that itself cannot be completely explained through a quantitative analysis. This calls for a qualitative or mixed method study that is able to dive deeper into the precise mechanisms behind these interactions.

## 6 Conclusion

The findings alluded to the possibility that improvement in loneliness levels is possible by promoting the use of DCT to counter loneliness, imposed by stay-at-home or social distancing orders associated with Covid-19, future pandemics or crises, or even to combat general isolation and loneliness in older adults. Tools such as DCT including the use of generative artificial intelligence (Generative AI) can be integrated into crisis communications, public health responses, and care programs to address loneliness among older adults, and helping them obtain information that would improve resilience in face of difficulties. Taking these elements into consideration will help decision-makers to develop a strong, effective approach. We saw that technology also mediated the relationship between psychological resilience and loneliness. However, psychological resilience and technology did not have a significant moderating impact on the relationship between social isolation and loneliness, hinting at other factors at play and a complex layered picture that needs further investigation.

The research infers that screening of older adults for psychological resilience levels and low technology experience may help identify those most at risk for adapting poorly when exposed to crises such as pandemics and wars. To this end, early interventions can help to build resilience among this demographic.

## Data Availability

The raw data supporting the conclusion of this article will be made available by the authors, without undue reservation.
